# Knowledge, attitudes and practices (KAP) toward artificial intelligence in rehabilitation among occupational therapists: A cross-sectional online survey in Korea

**DOI:** 10.1097/MD.0000000000045701

**Published:** 2025-11-07

**Authors:** Chun-Yeop Lee, Nam-Hae Jung

**Affiliations:** a Department of Occupational Therapy, Kaya University, Gimhae, Republic of Korea; b Department of Occupational Therapy, Dongseo University, Busan, Republic of Korea.

**Keywords:** artificial intelligence (AI), knowledge, attitudes and practices (KAP), knowledge, Korea, occupational therapists

## Abstract

In the healthcare field, artificial intelligence (AI) enhances diagnostic precision, optimizes treatment strategies, and enables tailored care delivery. The attitudes and acceptance of AI among healthcare professionals critically determine the rate of technological integration and practical application. This study investigates occupational therapists’ knowledge, attitudes, and practices concerning AI in rehabilitation settings, while analyzing associated influencing factors. An online survey was administered to 224 occupational therapists to evaluate their knowledge, attitudes, and practices of AI, alongside their future adoption intentions. The study further explored demographic-based disparities in these variables and assessed the relationship between each factor and adoption intent. Higher educational levels were associated with greater knowledge, more positive attitudes, and higher engagement in AI-related practices. Importantly, attitudes played a key mediating role: while knowledge and practice did not directly predict adoption intentions, both influenced intentions indirectly through attitudes. While occupational therapists generally exhibit optimism toward AI, institutional barriers – particularly insufficient organizational support and ethical frameworks – are perceived as major impediments to implementation. Effective AI adoption necessitates systemic changes, including standardized protocols, interdisciplinary training programs, and policy reforms, complementing individual mindset shifts.

## 1. Introduction

Artificial intelligence (AI) refers to the mimicry and problem-solving capabilities that human intelligence exhibits in machines, which are specifically designed and programmed to perform tasks typically requiring human intelligence, such as sensing, thinking, learning, and decision-making.^[1,2]^ AI has served as a crucial tool for the advancing healthcare technology. From a healthcare perspective, AI drives a paradigm shift in the field, fueled by the growing availability of healthcare data and rapid progress in analytics technologies.^[3]^ Today, AI is actively applied to enhance diagnostic accuracy, optimize treatment planning, and improve various facets of patient care.^[4]^

AI is well-suited to automating repetitive workflows, processing vast datasets, and minimizing errors in clinical decision-making.^[5]^ Furthermore, it plays a critical role in delivering real-time alerts to patients and clinicians, improving diagnostic precision, and facilitating individualized therapeutic strategies.^[6,7]^ Patients in underserved regions with limited access to healthcare providers can benefit from remote diagnosis and treatment.^[8,9]^ Current applications span diverse medical specialties, such as diagnostic imaging, pathology, ophthalmology, and cardiology, where AI tools are increasingly integrated into clinical practice.^[5,10–12]^

In rehabilitation, AI has enhanced the patient treatment process by providing comprehensive assessments, predicting spasticity scores via wearable arm sensors, and forecasting patient outcomes.^[13,14]^ Additionally, exoskeleton robots have been employed to improve gait in stroke patients and facilitate rehabilitation, while social assistive robots have been utilized to promote social interaction among homebound elderly individuals, alleviate loneliness, and enhance their well-being.^[15,16]^ In occupational therapy, AI assists clients in progressing through treatment by reminding them of occupational goals and strategies for achieving those objectives.^[17]^ Furthermore, AI has predicted compliance with cognitive training programs and can automatically collect data on the occupations performed by clients, generating a calendar view that enables occupational therapists and clients to discuss occupational balance.^[18,19]^

As AI is increasingly integrated into the medical industry, the role of healthcare professionals has become even more critical.^[20]^ The attitudes and acceptance levels of these professionals toward AI significantly influence the pace of technological adoption and its practical implementation. Studies investigating knowledge, attitudes, and practices related to AI have been conducted across diverse healthcare disciplines, including physicians, nurses, pharmacists, physical therapists, and radiologists.^[21–24]^ However, occupational therapists have received far less research attention, despite their unique role in rehabilitation and client-centered practice. This gap limits our understanding of how AI adoption may unfold within occupational therapy. While most professionals understood the general concept of AI, their familiarity with specific AI applications remained limited. They acknowledged the importance of AI in healthcare and emphasized the need for specialized training, yet reported minimal hands-on experience with its use. Concerns also emerged regarding potential job displacement due to AI. Notably, these findings revealed substantial various based on demographic factors such as gender, educational background, and professional experience, highlighting the presence of multifaceted barriers to AI adoption. Consequently, targeted educational initiatives, skill development programs, and financial incentives are deemed essential to facilitate broader integration.

In occupational therapy, AI holds potential to offer benefit; however, its implementation may also pose risks and challenges tied to the ethical principles underpinning the discipline.^[17]^ For instance, core therapeutic components such as empathetic engagement derived from lived experiences and the intentional use of the therapist’s self as a therapeutic tool remain uniquely human capacities that AI cannot fully replicate or comprehend.^[25]^ Consequently, maintaining recognition of occupational therapists’ irreplaceable value and role emerges as a crucial factor for successful AI integration. Nevertheless, research specifically examining occupational therapists’ knowledge, attitudes, and practices regarding AI remains limited. This study seeks to address this gap by situating occupational therapists within the broader discourse on AI adoption and providing novel insights that are distinct from prior studies conducted with other healthcare professionals.

This study seeks to assess the level of knowledge, attitudes, and practices among occupational therapists regarding AI and to investigate the factors influencing these dimensions. The findings may serve as foundational data for developing AI-integrated educational frameworks within future occupational therapy curricula, formulating strategies to advance digital healthcare applications, and enhancing systemic acceptance of emerging technologies. The hypotheses posited in this study are as follows:

1. Occupational therapists’ knowledge, attitudes, practices, and intentions toward AI will vary depending on their general characteristics.

2. There will be a significant correlation among occupational therapists’ knowledge, attitudes, practices, and intentions toward AI.

3. Occupational therapists’ knowledge, attitudes, and practices will influence their intentions to use AI.

## 2. Materials and methods

### 2.1. Study design

A cross-sectional survey study was conducted to assess occupational therapists’ knowledge, attitudes, and practices in Korea regarding AI utilization in rehabilitation. Data were collected from June 24 to July 16, 2025. The study received approval from the Institutional Review Board at Kaya University (KAYA IRB-425) and adhered to the ethical principles outlined in the Declaration of Helsinki.

### 2.2. Participants and data collection

The appropriate sample size for this study was calculated using the G*Power program (version 3.1, Heinrich Heine University Düsseldorf, Düsseldorf, Germany). The effect size was set at 0.50, the significance level (α) at 0.05, and the statistical power (1-β) at 0.95. Based on these parameters, the minimum required sample size was determined to be 176. A total of 226 participants completed the survey, and data from 224 individuals – excluding 2 participants who did not consent to the collection of personal information – were included in the final analysis. This demonstrates that the achieved sample size sufficiently exceeded the calculated minimum, ensuring adequate statistical power to detect the preset effect size (0.50).

In addition, the final sample size of 224 meets the recommended thresholds for structural equation modeling (SEM). According to previous guidelines, models with moderate complexity involving multiple latent and observed variables generally require a minimum of 200 participants to ensure reliable parameter estimation and model stability.^[26]^ Therefore, the sample size was deemed adequate for both regression and SEM analyses conducted in this study.

The sample was selected through convenience sampling techniques. The survey was administered online via Google Forms to participants who fully understood the study’s objectives, methodology, and provided voluntary informed consent. The researcher shared the survey link on social media platforms frequented by licensed occupational therapists in practice. To uphold participant confidentiality, all responses were anonymized during data collection.

The KAP questionnaire utilized in this study was developed by the researchers, drawing on prior literature.^[4,9,21,23,27]^ The questionnaire comprises 50 items across 4 domains: 6 questions on the general characteristics of participants, 6 questions on knowledge of AI, 22 questions on attitudes toward AI (14 positively framed, 8 negatively framed), and 16 questions on practice and intentions to use AI. Except for the items on AI practice and intention to use, all questions were structured as closed-ended items rated on a 5 point Likert scale, ranging from “strongly agree” (5 points) to “strongly disagree” (1 point). The items related to AI practice and intention to use comprised 16 specific topics, each requiring participants to respond with a “Yes” or “No” to indicated whether they had experience with the given AI application and whether they intended to use it. To ensure the content validity, the instrument underwent rigorous evaluation by 5 experts, resulting in a content validity ratio of ≥0.80 for all items. Furthermore, a pilot test was conducted with 30 individuals not involved in the main study, and the internal consistency (Cronbach alpha) was 0.84 for knowledge, 0.89 for attitude, 0.94 for practice, and 0.93 for intentions.

### 2.3. Theoretical framework

The Knowledge, Attitude, and Practice (KAP) model is the most widely adopted theoretical framework for investigating how an individual’s knowledge and beliefs influence health behavior change.^[28]^ This model conceptualizes behavioral transformation across 3 sequential stages: knowledge acquisition, attitudinal belief formation, and behavioral adoption. KAP research employs a systematic and empirically grounded methodology, primarily relying on tailored questionnaires designed to align with specific research objectives and target populations.^[29]^ While traditionally utilized in health sciences, the KAP framework has demonstrated adaptability to emerging contexts, such as the integration of AI into therapeutic practices.^[30]^ This questionnaire consists of a title, introduction, sociodemographic background, and questions about KAP. Data is collected through self-report or interviews.^[30,31]^ Analysis of the questionnaire provides comparative group studies, interventions, and practical recommendations.^[32]^ By systematically mapping cognitive, affective, and behavioral dimensions, the KAP model offers a robust tool for understanding and promoting sustainable health behavior changes in evolving technological landscapes.

### 2.4. Data analysis

This study utilized IBM SPSS Statistics 28.0 and Amos 26.0 (IBM Corporation, Armonk) for data analysis. The significance level (α) was set at 0.05 for all statistical tests. Descriptive statistical analysis (e.g., frequency, percentage, mean, and standard deviation) were conducted to characterize the sample demographics and assess participant’s knowledge, attitude, and practice levels. A normality test was performed on key variable scores using the Kolmogorov–Smirnov and Shapiro–Wilk tests. Most variables demonstrated *P*-values < .05, violating the assumption of normality. Consequently, the following nonparametric methods were adopted for subsequent analyses. The Mann–Whitney *U* test was employed to evaluate gender-based differences in variable scores, while the Kruskal–Wallis test assessed disparities across educational levels. To mitigate inflated Type I error rates during post hoc comparisons, the Bonferroni correction was systematically applied. Additionally, Spearman rank correlation coefficient (ρ) was utilized to examine monotonic associations between variables. A multiple regression framework subsequently identified key predictors of AI adoption intent. Finally, SEM was conducted to empirically validate hypothesized relationships among latent constructs. The SEM model’s goodness-of-fit was rigorously assessed through multiple indices: Chi-square (χ²) was deemed acceptable when the *P*-value exceeded .05. Comparative fit index and Tucker-Lewis index indicated adequate fit at values ≥ 0.90. Goodness-of-fit index reached adequacy at thresholds ≥ 0.90. Root mean square error of approximation demonstrated satisfactory performance at ≤0.08, while superior fit was marked at ≤0.05.^[33,34]^

## 3. Results

### 3.1. General characteristics of participants

A total of 224 occupational therapists participated in the study. The majority were female, with participants distributed across their 20s to 50s and older. About one-third had <5 years of clinical experience, and half held a bachelor’s degree. Participants represented diverse practice settings and client populations (Table 1).

**Table 1:** General characteristics of participants (n = 224).

Variables	Categories	Frequency (n)	Percentage (%)
Sex	Man	82	36.7
	Woman	142	63.3
Age (yr)	20s	69	30.8
	30s	79	35.3
	40s ≥ 50s	45 31	20.1 13.8
	M ± SD	34.35 ± 6.90	
Clinical experience (yr)	≤ 5	73	32.6
	6–10	65	29.0
	11–15	43	19.2
	≥ 16	43	19.2
	M ± SD	9.16 ± 6.17	
Educational level	Associate	40	17.9
	Bachelor	112	50.0
	Master	50	22.3
	Doctorate	22	9.8
Type of institutions	Hospital	182	81.3
	Private center for children	35	15.6
	Long-term care facility	20	8.9
	Public health center	35	15.6
	Welfare center	20	8.9
	Educational institution	19	8.5
	Mental health facility	17	7.6
	Child care center	10	4.5
Client populations	Adults and elderly	199	88.8
	Children	104	46.4
	Individuals with mental health conditions	36	16.1

M ± SD = mean ± standard deviation.

### 3.2. Knowledge toward AI

Most participants reported a general understanding of AI and had obtained related information through online sources. However, awareness of specific applications in healthcare and rehabilitation was low, and only a small proportion had received formal education or engaged in AI-related research (Table 2).

**Table 2:** Knowledge toward AI, N (%).

Items	Strongly agree	Agree	Neutral	Disagree	Strongly disagree	M ± SD
1. I understand what AI is.	62 (27.7)	94 (42.0)	60 (26.8)	6 (2.7)	2 (0.9)	3.92 ± 0.85
2. I know examples of AI applications in the healthcare field.	16 (7.1)	44 (19.6)	63 (28.1)	49 (21.9)	52 (23.2)	2.65 ± 1.23
3. I know examples of AI applications in the rehabilitation field.	16 (7.1)	25 (11.2)	51 (22.8)	62 (27.7)	70 (31.3)	2.55 ± 1.22
4. I have learned about AI through school or professional education.	6 (2.7)	13 (5.8)	18 (8.0)	53 (23.7)	134 (59.8)	1.67 ± 1.02
5. I have obtained AI-related information through the news or online sources.	72 (32.1)	83 (37.1)	50 (22.3)	14 (6.3)	5 (2.2)	3.02 ± 0.99
6. I have participated in research related to AI.	5 (2.2)	10 (4.5)	7 (3.1)	14 (6.3)	188 (83.9)	1.02 ± 0.91

AI = artificial intelligence, M ± SD = mean ± standard deviation.

### 3.3. Attitude toward AI

Overall, participants expressed positive attitudes toward AI. The majority believed that AI could be applied in practice with sufficient training and that it would be beneficial in healthcare. In contrast, many felt that their work environments did not yet support AI adoption. Concerns were also noted regarding insufficient regulations and the need for human supervision, whereas most disagreed with the idea that AI weakens therapist–client relationships (Table 3).

**Table 3:** Attitude toward AI. N (%).

Items	Strongly agree	Agree	Neutral	Disagree	Strongly disagree	M ± SD
1. I believe that AI is beneficial in the healthcare field.	86 (38.4)	91 (40.6)	39 (17.4)	7 (3.1)	1 (0.4)	4.13 ± 0.84
2. I believe that AI is beneficial in the rehabilitation field.	67 (29.9)	97 (43.3)	48 (21.4)	11 (4.9)	1 (0.4)	3.97 ± 0.87
3. I believe that AI is helpful during client evaluation.	56 (25.0)	104 (46.4)	47 (21.0)	13 (5.8)	4 (1.8)	3.91 ± 0.86
4. I believe that AI is helpful in goal setting and treatment planning.	60 (26.8)	108 (48.2)	39 (17.4)	15 (6.7)	2 (0.9)	3.95 ± 0.88
5. I believe that AI is helpful in intervention implementation.	54 (24.1)	96 (42.9)	57 (25.4)	13 (5.8)	4 (1.8)	3.91 ± 0.88
6. I believe that if sufficient training is provided, I can apply AI in clinical practice.	74 (33.0)	107 (47.8)	34 (15.2)	7 (3.1)	2 (0.9)	3.93 ± 0.85
7. I believe that if I have enough time, I can learn and apply AI technologies myself.	72 (32.1)	98 (43.8)	41 (18.3)	10 (4.5)	3 (1.3)	4.10 ± 0.90
8. I feel that my work environment (e.g., hospital, institution) supports the application of AI.	25 (11.2)	28 (12.5)	44 (19.6)	66 (29.5)	61 (27.2)	3.99 ± 0.89
9. I am willing to actively adopt AI technologies in my work.	63 (28.1)	90 (40.2)	58 (25.9)	9 (4.0)	4 (1.8)	3.83 ± 1.31
10. I believe AI can help reduce the workload of occupational therapists.	51 (22.8)	74 (33.0)	55 (24.6)	33 (14.7)	11 (4.9)	3.92 ± 0.93
11. I believe AI can reduce errors and increase accuracy in the occupational therapy process.	45 (20.1)	78 (34.8)	73 (32.6)	23 (10.3)	5 (2.2)	3.96 ± 0.92
12. I believe AI can provide useful and relevant information for therapy.	64 (28.6)	87 (38.8)	59 (26.3)	10 (4.5)	4 (1.8)	4.01 ± 0.88
13. I believe AI can improve treatment accessibility for a greater number of clients.	57 (25.4)	83 (37.1)	64 (28.6)	15 (6.7)	5 (2.2)	4.10 ± 0.87
14. I believe AI can reduce the financial burden of institutional operations.	35 (15.6)	58 (25.9)	76 (33.9)	35 (15.6)	20 (8.9)	3.92 ± 0.91
15*. I am concerned that AI may eventually replace the role of occupational therapists.	25 (11.2)	31 (13.8)	44 (19.6)	59 (26.3)	65 (29.0)	2.51 ± 1.33
16*. I believe that healthcare laws and regulations related to AI are insufficient.	79 (35.3)	86 (38.4)	47 (21.0)	7 (3.1)	5 (2.2)	4.01 ± 0.94
17*. I believe AI could become a burden for occupational therapists.	8 (3.6)	36 (16.1)	62 (27.7)	80 (35.7)	38 (17.0)	2.53 ± 1.06
18*. I believe the use of AI could lead to serious privacy issues.	40 (17.9)	74 (33.0)	70 (31.3)	34 (15.2)	6 (2.7)	3.48 ± 1.03
19*. I am uncertain about the outcomes of AI errors and worry about its safety.	40 (17.9)	84 (37.5)	70 (31.3)	26 (11.6)	4 (1.8)	3.58 ± 0.97
20*. I believe AI lacks flexibility and has limited ability to empathize with and consider clients’ emotional well-being.	45 (20.1)	89 (39.7)	54 (24.1)	28 (12.5)	8 (3.6)	3.60 ± 1.05
21*. I believe AI weakens the relationship between clients and occupational therapists.	7 (3.1)	25 (11.2)	58 (25.9)	92 (41.1)	42 (18.8)	2.38 ± 1.01
22*. I believe AI requires human supervision.	126 (56.3)	70 (31.3)	21 (9.4)	5 (2.2)	2 (0.9)	4.39 ± 0.81

AI = artificial intelligence, M ± SD = mean ± standard deviation.

* Items 15 to 22 were negatively worded.

### 3.4. Practical experience and intention to use toward AI

Practical experience with AI tools was limited, with ChatGPT being the most commonly used. Nevertheless, intention to use AI in the future was high across a wide range of applications, particularly for AI-based training programs and knowledge recommendation systems (Table 4).

**Table 4:** Practical experience and intention to use toward AI, N (%).

Items	Practical experience	Intention to use
	Yes	Yes
1. Exploring occupational therapy information using ChatGPT	154 (68.8)	195 (87.1)
2. AI-based tailored knowledge recommendation system for therapists	91 (40.6)	196 (87.5)
3. AI assessment system for early prediction of cognitive decline	42 (18.8)	185 (82.6)
4. Real-time analysis of client movements	42 (18.8)	175 (78.1)
5. Automated generation tool for occupational therapy SOAP notes	35 (15.6)	174 (77.7)
6. Tailored intervention plan recommendation system	48 (21.4)	179 (79.9)
7. Predictive analysis system for therapy progress and prognosis	32 (14.3)	186 (83.0)
8. AI-based visual and attention training programs	48 (21.4)	198 (88.4)
9. VR or AR-based immersive occupational therapy training systems	77 (34.4)	181 (80.8)
10. Robot-assisted occupational therapy support systems	59 (26.3)	188 (83.9)
11. Response-based feedback systems for sensory integration therapy	26 (11.6)	165 (73.7)
12. AI-supported remote occupational therapy services at home	27 (12.1)	173 (77.2)
13. Self-therapy and monitoring platforms through mobile applications	45 (20.1)	173 (77.2)
14. AI voice analysis technology for detecting emotional states and depression risk	26 (11.6)	162 (72.3)
15. AI-assisted administrative documentation in occupational therapy	41 (18.3)	195 (87.1)
16. AI communication interface to support client–therapist relationship	22 (9.8)	165 (73.7)

AI = artificial intelligence, AR = augmented reality, GPT = generative pre-trained transformer, SOAP = subjective, objective, assessment, and plan, VR = virtual reality.

### 3.5. Differences in AI knowledge, attitude, practice, and intention according to gender and educational level

Male therapists reported significantly more positive attitudes toward AI compared with females, while other domains showed no gender differences. Educational level was associated with significant differences: those with higher degrees, especially doctorates, reported greater knowledge, more positive attitudes, and higher engagement in AI practices. No differences were observed for intention to use (Tables 5 and 6).

**Table 5:** Differences in AI knowledge, attitude, practice, and intention according to gender.

Variable	Male M ± SD	Female M ± SD	*P*-value
AI knowledge	16.1 ± 4.2	15.7 ± 4.1	.481
AI attitude	75.6 ± 10.7	72.7 ± 10.6	.040
AI practice	4.1 ± 4.6	3.3 ± 4.1	.221
AI intention to use	12.3 ± 4.9	13.2 ± 4.0	.259

AI = artificial intelligence, M ± SD = mean ± standard deviation.

**Table 6:** Differences in AI knowledge, attitude, practice, and intention according to educational level.

Variable	M ± SD	Chi-square	*P*-value (post hoc)
AI knowledge			
Associate^a^	14.52 ± 4.29	32.99	<.001 (d > a,b,c)
Bachelor^b^	15.19 ± 3.39		
Master^c^	16.20 ± 3.92		
Doctorate^d^	21.00 ± 4.22		
AI attitude			
Associate^a^	68.92 ± 9.48	16.28	.001 (b,d > a)
Bachelor^b^	74.07 ± 10.99		
Master^c^	74.42 ± 11.28		
Doctorate^d^	79.18 ± 7.05		
AI practice			
Associate^a^	2.75 ± 4.27	13.58	.004 (c,d > a)
Bachelor^b^	3.41 ± 4.27		
Master^c^	4.44 ± 4.54		
Doctorate^d^	4.54 ± 4.19		
AI intention to use			
Associate^a^	11.80 ± 5.49	2.10	.552
Bachelor^b^	12.68 ± 4.66		
Master^c^	13.88 ± 2.71		
Doctorate^d^	13.95 ± 3.59		

AI = artificial intelligence, M ± SD = mean ± standard deviation.

### 3.6. Correlations among AI knowledge, attitude, practice, and intention

Correlation analysis revealed that knowledge was positively associated with both attitude and practice, but not directly with intention. Attitude showed a moderate association with intention, suggesting its central role in AI adoption. Practice was weakly associated with attitude but not with intention (Table 7).

**Table 7:** Correlations among AI knowledge, attitude, practice, and intention to use.

	AI knowledge	AI attitude	AI practice	AI intention to use
AI knowledge	1			
AI attitude	.290**	1		
AI practice	.279**	.226**	1	
AI intention	.078	.424**	.102	1

AI = artificial intelligence, *r*_s_ = Spearman *r*.

* *P* < .05.

** *P* < .01.

### 3.7. Multiple regression analysis for predicting AI usage intention

Regression results showed that only attitude was a significant predictor of intention to use AI, whereas knowledge and practice were not. The overall explanatory power of the model was modest. No multicollinearity or autocorrelation issues were identified (Table 8).

**Table 8:** Multiple regression analysis for factors associated with intention to use AI.

Variable	B	SE B	β	*t*	*P*	VIF
(Constant)	−0.433	2.137		−0.203	.840	
AI knowledge	−0.0009	0.076	−0.008	−0.118	.906	1.35
AI attitude	0.174	0.026	0.424	6.572	<.001	1.42
AI practice	0.022	0.068	0.021	0.322	.748	1.33
Gender	1.336	0.564	0.146	2.369	.019	1.18
Age	−0.024	0.010	−0.148	−2.331	.021	1.27
Clinical experience	0.000	0.004	−0.004	−0.062	.950	1.15
Bachelor’s degree	0.277	0.746	0.031	0.371	.711	1.09
Master’s degree	1.565	0.882	0.148	1.775	.077	1.16
Doctorate degree	1.326	1.179	0.090	1.125	.262	1.20

AI = artificial intelligence, SE = standard error, VIF = variance inflation factors, β = standardized regression coefficient.

### 3.8. SEM analysis for explore the structural relationships

**Figure 1.**
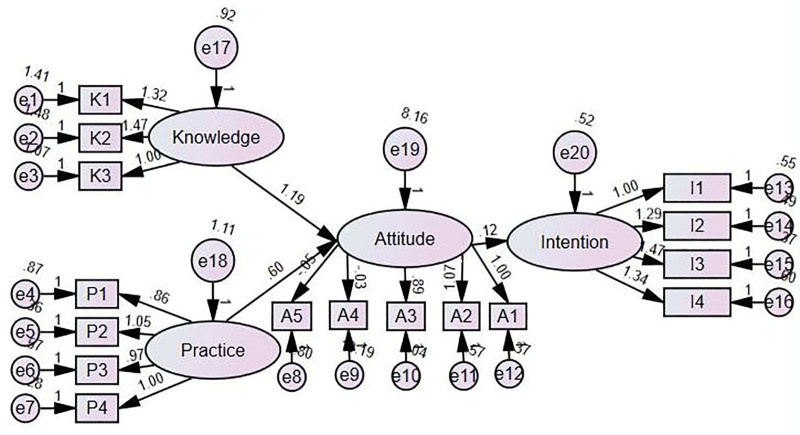
Structural equation model of the relationships among knowledge, practice, attitude, and intention to use.

Building on these regression results, which highlighted the central role of attitude, a structural equation model was employed to further examine whether knowledge and practice influenced intention indirectly through attitudes. The model demonstrated acceptable fit, and path analysis confirmed that both knowledge and practice influenced intention indirectly through attitude. These findings underscore the mediating role of attitude in AI adoption among occupational therapists (Table 9, Fig. 1).

**Table 9:** SEM results.

Path	β	SE	CR	*P*	Sig.
Attitude ← Knowledge	1.187	0.273	4.354	<.001	Significant
Attitude ← Practice	0.601	0.210	2.859	.004	Significant
Intention ← Attitude	0.119	0.020	5.997	<.001	Significant

CR = critical ratio, SE = standard error, SEM = structural equation modeling, β = standardized regression coefficient.

## 4. Discussion

This study explored the effects of occupational therapists’ knowledge, attitude, and practice of AI on their intention to use AI, and examined the causal pathways among these factors using a structural equation model. Overall, while the attitude of AI among occupational therapists was relatively positive, both AI-related knowledge and practice were limited. These findings are consistent with previous studies reporting that although healthcare professionals express a generally favorable attitude toward AI, their practical experience and technical knowledge remain insufficient.^[35,36]^ International studies among occupational therapists in Malaysia also identified similar challenges: optimism about AI’s potential coexists with concerns regarding the rapid pace of technological advancement and inadequate clinical readiness or educational support.^[35]^

Republic of Korea literature also highlights the opportunities and challenges posed by AI in occupational therapy. A systematic review by Shin and Cho emphasized that although AI holds promise for enhancing decision-making and data-driven interventions, the lack of institutional frameworks and ethical guidelines may hinder practical implementation.^[37]^ Likewise, undergraduate research by Kim et al revealed positive expectations for AI applications, such as efficiency and objectivity, but also raised concerns about job security and the disruption of therapeutic relationships.^[38]^ Additionally, Koo noted that in the context of the Fourth Industrial Revolution, occupational therapists must redefine their roles by embracing interdisciplinary competencies and adapting to technological shifts.^[39]^ These perspectives align with the present study’s findings, suggesting that the key to successful AI adoption lies not solely in knowledge acquisition, but also in shifting professional identity and fostering institutional support.

Gender- and education-related differences were also observed. Male therapists reported significantly more favorable attitudes toward AI, although female therapists tended to show higher intention to use AI, suggesting potential gaps between perception and behavioral intention. Participants with doctoral degrees scored significantly higher in knowledge, attitude, and experience, implying that educational attainment may facilitate AI-related readiness, and tailored education strategies are warranted. These findings emphasize that interventions should not only aim to enhance factual knowledge but also integrate AI-related competencies into occupational therapy curricula and continuing professional development programs. Embedding AI literacy, ethical considerations, and applied training opportunities within education and professional training will be critical to ensuring that occupational therapists are prepared to adopt and implement AI effectively in practice.

Correlation analyses revealed that AI attitude was significantly and positively correlated with AI knowledge, experience, and intention to use, with a moderate correlation with intention, underscoring the central role of attitude in AI adoption. In contrast, AI knowledge and experience did not show significant direct correlations with intention, suggesting a possible mediating effect through attitude. These findings emphasize the need for interventions that go beyond factual education, instead focusing on fostering positive attitudes.

Multiple regression analysis further supported these results, showing that only AI attitude (β = 0.424, *P* < .001) significantly predicted intention to use AI, while knowledge and experience were not significant predictors. However, in the SEM model, both AI knowledge and experience indirectly influenced intention through attitude. This supports the Technology Acceptance Model, which posits that perceived usefulness and attitude are critical mediators in technology adoption.^[40]^

Survey results reinforced the pivotal role of attitude. A total of 80.8% of participants agreed with the statement, “I believe that if sufficient training is provided, I can apply AI in clinical practice,” and 79.0% viewed AI as beneficial in healthcare. This suggests that therapists are willing to accept AI when proper education is provided, rather than perceiving it as a threat. However, 56.7% reported that their work environments did not support AI usage, and many expressed concerns regarding insufficient legal frameworks (73.7%) and the necessity of human oversight (87.6%). Notably, 59.9% disagreed with the notion that “AI weakens the client-therapist relationship,” indicating that AI is largely viewed as a supportive tool rather than a substitute. These findings support the view that the successful adoption of AI in occupational therapy is influenced more by environmental and institutional conditions than by individual resistance. Castagno and Khalifa highlighted that, even when healthcare professionals exhibit positive attitudes toward AI, its implementation is often hindered by the absence of regulatory clarity, ethical accountability, and sufficient organizational support.^[41]^ These results suggest that overcoming systemic and structural barriers may be more critical than changing individual perceptions alone when promoting AI adoption in rehabilitation settings.

Despite the significance of attitude, the explanatory power of the regression model was modest (*R*^2^ = 10.8%, adj. *R*^2^ = 7.5%), suggesting that additional factors beyond individual knowledge and attitude – such as workplace culture, accessibility of AI tools, institutional policies, and systemic support – may play a substantial role in shaping AI adoption. Future research should incorporate these structural and organizational variables to provide a more comprehensive understanding of the mechanisms underlying technology acceptance in occupational therapy. This study contributes to a growing body of evidence on AI readiness in occupational therapy, providing empirical insights to inform future interventions, curricula, and policy development. As the profession navigates the evolving landscape of AI, this research underscores the importance of a balanced approach that cultivates technological competencies while preserving client-centered values.

### 4.1. Study limitations

This study has several limitations. First, its cross-sectional design limits the ability to draw causal inferences between variables. Second, the use of self-reported questionnaires may have introduced social desirability bias, particularly in responses related to attitudes and intentions toward AI. Third, the study was conducted among occupational therapists in the Republic of Korea, which may limit the generalizability of the findings to other countries or professional groups. Fourth, the use of convenience sampling through social media may have biased the sample toward therapists with higher digital literacy or stronger interest in technology, which further limits generalizability. Fifth, some survey items (e.g., AI practice and intention to use) were measured in a binary format, which may have oversimplified complex behaviors and reduced the nuance of participants’ responses. Sixth, some demographic items, such as workplace settings and client populations, allowed multiple responses, which limited subgroup comparisons and cross-tabulation analyses. Lastly, structural factors such as organizational culture and institutional support were not included in the analysis, which may have constrained a more comprehensive understanding of AI adoption.

## 5. Conclusions

This study investigated factors associated with occupational therapists’ knowledge, attitudes, practices, and intentions toward AI applications in rehabilitation settings. The findings demonstrated that higher educational attainment was positively correlated with elevated levels of knowledge, more favorable attitudes toward AI, and greater engagement in AI-related practices. Mediation analysis further revealed that attitudes partially mediated the relationships between knowledge/practice and adoption intentions, while attitudes themselves exerted a direct predictive influence on AI adoption intentions. For the successful adoption of AI in the future, standardized protocols, interdisciplinary continuing educational programs, policy reform, and targeted awareness initiatives are essential. In particular, professional associations and educational institutions should play a leading role in integrating AI-related education and competencies into occupational therapy training and continuing professional development, thereby bridging current knowledge gaps and strengthening readiness for AI adoption in practice.

## Author contributions

**Conceptualization:** Chun-Yeop Lee, Nam-Hae Jung.

**Data curation:** Chun-Yeop Lee, Nam-Hae Jung.

**Formal analysis:** Chun-Yeop Lee, Nam-Hae Jung.

**Investigation:** Chun-Yeop Lee, Nam-Hae Jung.

**Methodology:** Chun-Yeop Lee.

**Project administration:** Chun-Yeop Lee, Nam-Hae Jung.

**Supervision:** Chun-Yeop Lee.

**Validation:** Chun-Yeop Lee.

**Visualization:** Chun-Yeop Lee.

**Writing – original draft:** Chun-Yeop Lee.

**Writing – review & editing:** Nam-Hae Jung.
